# Thyroid surgery – intraoperative localization and protection of important structures of the neck

**DOI:** 10.1186/1756-6614-6-S2-A12

**Published:** 2013-04-05

**Authors:** Jan Brzeziński

**Affiliations:** 1 Department of General, Oncological and Endocrine Surgery, Medical University of Lodz, Poland; 2 Polish Mother’s Memorial Hospital – Research Institute, Lodz, Poland

## 

Nodular thyroid disease affects 500 to 600 million people worldwide. Tumours of the thyroid account for about 1% overall human cancers. Thyroidectomy is the most common endocrine operation. Surgical treatment for benign thyroid nodules is recommended for: progressive increase in nodule size, substernal extension, compressive symptoms in the neck region, the development of thyrotoxicosis and in case of preference of that kind of treatment reported by the patient. In Poland thyroidectomy is the fourth surgical procedure and concerns 25000 operations yearly. Reduction of surgical injury with simultaneous retention of current safety and radical nature of surgical procedure forces the work in a relatively small operating field. Electric devices enabling the achievement of full and lasting haemostasis during thyroidectomy supplant traditional surgical method (ligature, haemostatic sutures) with no impact on the incidence of perioperative complications, while at the same time allowing to shorten the duration of the procedure. The haemostatic effect is associated with generation of heat, which apart from the intended result may bring about thermal tissue injury. During the surgical procedure important is to determine the thermal spread around the active tip of electric devices in the operating field during thyroidectomy, and the safe temperature range during the operation to protect important structures of the neck. The mean safe distance of the active tip of an electric device from important anatomic structures is 5mm minimally and depends on the device type, time of using and its power settings. All the modern techniques of vessel sealing are associated with generation of heat and its spherical spread, which causes thermal injury to the surrounding tissues. Their mode of operation through, among others, structural changes in collagen and elastin, leads to lasting joining of sealed vessel walls and tissue structures. These systems enable a safe sealing of vessels of up to 7mm in diameter.

In conclusions: In the cases analysed by the author concerning the thyroidectomy techniques, it is recommended to replace to electric devices with ligatures or clips or human fibrinogen in place near the laryngeal nerves, parathyroid glands and the trachea. The decision on the change of the method of haemostasis maintenance in the vicinity of crucial structures belongs to the surgeon.

